# Exosome-Derived microRNAs as Liquid-Biopsy Biomarkers in Laryngeal Squamous Cell Carcinoma: A Narrative Review and Evidence Map

**DOI:** 10.3390/biomedicines13122929

**Published:** 2025-11-28

**Authors:** Crina Oana Pintea, Cristian Ion Mot, Islam Ragab, Şerban Talpoş, Karina-Cristina Marin, Nicolae Constantin Balica, Edward Seclaman, Kristine Guran, Delia Ioana Horhat

**Affiliations:** 1 Doctoral School, “Victor Babes” University of Medicine and Pharmacy Timisoara, Eftimie Murgu Square 2, 300041 Timisoara, Romania; crina.pintea@umft.ro (C.O.P.); guran.kristine@umft.ro (K.G.); 2 Department IX, Discipline of Otolaryngology, “Victor Babes” University of Medicine and Pharmacy Timisoara, Eftimie Murgu Square 2, 300041 Timisoara, Romania; balica@umft.ro (N.C.B.); horhat.ioana@umft.ro (D.I.H.); 3 Faculty of Medicine, Misr University for Science & Technology, Giza Governorate 3237101, Egypt; islamayman772@gmail.com; 4 Discipline of Oral and Maxillo-Facial Surgery, Faculty of Dental Medicine, “Victor Babes” University of Medicine and Pharmacy Timisoara, Eftimie Murgu Square 2, 300041 Timisoara, Romania; 5 Department of Biochemistry and Pharmacology, “Victor Babes” University of Medicine and Pharmacy Timisoara, Eftimie Murgu Square 2, 300041 Timisoara, Romania; marin.karina@umft.ro (K.-C.M.); eseclaman@umft.ro (E.S.)

**Keywords:** laryngeal squamous-cell carcinoma, extracellular vesicles, exosomes, maxillofacial surgery, microRNA, liquid biopsy, diagnostics, prognosis, tumour micro-environment

## Abstract

Exosome-derived microRNAs (miRNAs) have been proposed as minimally invasive biomarkers for laryngeal squamous- cell carcinoma (LSCC). Because oral and maxillofacial surgeons are integral to head-and-neck oncologic and reconstructive pathways, such liquid-biopsy signals could support perioperative decision-making (selection for organ-preserving surgery), margin surveillance, and reconstructive planning. We conducted a preregistered, protocol-driven search of PubMed/MEDLINE, Web of Science, and Scopus from inception to 1 June 2025. Given the very small number of clinically comparable diagnostic studies, discordant index tests/thresholds, and high heterogeneity, we did not perform quantitative pooling or publication-bias testing. Instead, we undertook a narrative synthesis and constructed an evidence map; risk of bias tools (QUADAS-2; ROBINS-I) were applied descriptively to inform qualitative confidence. Nine studies were formally analysed based on eligibility to the study topic. Two serum-based case–control investigations (111 LSCC, 80 controls) reported areas under the ROC curve of 0.876 (miR-21 + HOTAIR) and 0.797 (miR-941), with corresponding sensitivities of 94% and 82%. Seven mechanistic papers showed that vesicular cargos—including miR-1246, circPVT1, and LINC02191—drive STAT3-dependent M2 polarisation, NOTCH1-mediated stemness, Rap1b-VEGFR2 angiogenesis, and glycolytic re-programming, producing 1.6–2.6-fold increases in invasion, tube formation, or xenograft growth. Only three studies fulfilled MISEV-2018 characterisation criteria, and none incorporated external validation. This narrative review and evidence map identifies promising but preliminary diagnostic signals and biologically plausible mechanisms for exosomal miRNAs in LSCC; however, the evidence is sparse, single-region, methodologically inconsistent, and at high risk of bias. Findings do not support clinical implementation at this stage. Priorities include harmonised EV workflows, prespecified thresholds, and prospective, multi-centre validation.

## 1. Introduction

Laryngeal squamous cell carcinoma (LSCC) remains a stubborn public health challenge, with the most recent GLOBOCAN update estimating 184,615 new cases and 99,840 deaths worldwide in 2022—figures that have barely improved over the past decade despite advances in chemoradiotherapy [1]. In the United States, age-standardised incidence has plateaued at 2.5 per 100,000 persons, mirroring a levelling-off of smoking prevalence but still translating into ~13,000 new diagnoses each year [2]. Global-burden modelling further reveals striking geographic heterogeneity: Caribbean nations and parts of Eastern Europe record mortality rates more than double the world average, whereas East Asia—despite high incidence—shows comparatively lower case-fatality owing to earlier detection [3,4]. Such disparities reinforce the need for low-cost, population-wide screening tools capable of triaging high-risk individuals before symptomatic presentation.

Against this backdrop, extracellular vesicles (EVs)—particularly 30–150 nm exosomes—have emerged as ideal “mail carriers” of tumour molecular cargo. The MISEV-2018 consensus underscores their stability, ability to shield nucleic acids from RNase attack, and amenability to standardised isolation [5]. Compared with cell-free DNA (cfDNA), exosomal microRNAs (miRNAs) are more abundant in early-stage disease, require lower sequencing depth, and can be sampled from multiple biofluids—including saliva, which is especially attractive for laryngeal screening. Recent liquid-biopsy pipelines in lung cancer illustrate the clinical feasibility of exosome-based early detection, with multi-miRNA panels achieving areas under the curve (AUCs) exceeding 0.90 in prospective cohorts [6,7].

Head and neck oncology has quickly adopted this paradigm. Salivary exosomal miR-486-5p and miR-10b-5p discriminate oral and oropharyngeal cancers from benign mucosal lesions with AUC 0.83–0.88 [8], while nasopharyngeal carcinoma (NPC) studies show that exosomal miR-17-5p and miR-205-5p drive angiogenesis and correlate with metastasis and poor survival [9,10]. These findings highlight the dual diagnostic–mechanistic relevance of EV-borne miRNAs across anatomically adjacent malignancies and support their exploration in LSCC.

A second wave of research focuses on the tumour-immune interface. Meta-analyses of >60 cancer datasets reveal that exosomal miRNAs—including miR-1246, miR-934, and miR-106b-5p—skew macrophage polarisation toward an M2, tumour-supportive phenotype, thereby enhancing invasion and immune escape [11,12]. Such macrophage-remodelling roles may be particularly relevant for LSCC, where the superficial lamina propria harbours abundant resident macrophages capable of shaping early disease behaviour. Yet, aside from scattered cell-line experiments, LSCC-specific evidence remains scarce.

Despite enthusiasm, LSCC exosomal-miRNA studies are surprisingly absent. A 2022 scoping review of head-and-neck EV research noted “marked heterogeneity and low reproducibility” in LSCC datasets, with only 27% complying with MISEV reporting standards [13]. Concurrently, translational-science roadmaps emphasise that liquid-biopsy assays must demonstrate incremental value over fibre-optic laryngoscopy, offer cost-effectiveness in population screening, and withstand prospective validation [14,15].

Accordingly, this study was designed as a narrative review and evidence map focusing exclusively on exosome-derived miRNAs in LSCC. Our objectives were to (1) appraise study-level diagnostic performance, where available; (2) summarise mechanistic insights relevant to LSCC pathobiology; and (3) scrutinise methodological quality and identify evidence gaps preventing clinical translation. We prespecified quantitative synthesis (HSROC) only if ≥3 methodologically comparable studies evaluated the same index miRNA; otherwise, we planned a narrative synthesis and evidence mapping.

## 2. Search Methods

Methodological procedures for the literature search followed the PRISMA 2020 guidelines [16].

This narrative review adhered to the following PICO statement. Population: Adults with histologically confirmed LSCC. Index test/exposure: Biofluid-derived exosomal miRNAs quantified by qRT-PCR or sequencing. Comparator: Cancer-free controls or pre-/post-operative pairs for diagnostic accuracy; high vs. low expression for prognostic associations; appropriate negative/positive controls for mechanistic experiments. Outcomes: Sensitivity, specificity, AUC for diagnostic studies; OS/DFS (HRs) for prognostic studies; quantitative phenotypes (invasion fold-change, angiogenesis indices) for mechanistic work. Study designs: Case–control, cross-sectional, and prospective or retrospective cohort studies for diagnostic/prognostic objectives; in vitro or ex vivo functional experiments that utilised patient-derived vesicles for mechanistic objectives.

Exclusions: Tissue-only studies, narrative reviews, editorials, conference abstracts without full data, studies that did not confirm vesicle identity (no transmission electron microscopy, nanoparticle-tracking analysis, or CD63/CD81 Western blot), or reports not providing separate LSCC data when mixed with other head-and-neck subsites.

Three bibliographic databases—PubMed/MEDLINE, Web of Science Core Collection (Clarivate), and Scopus (Elsevier)—were searched from inception to 1 June 2025. No language, document-type, or publication-status restrictions were imposed. Reference lists of eligible papers and forward citations (via Google Scholar) were hand-searched. ClinicalTrials.gov and the WHO International Clinical Trials Registry Platform were screened for unpublished or ongoing studies.

Records were exported to EndNote and de-duplicated automatically. Two reviewers independently screened titles/abstracts and full texts in parallel. Disagreements were reconciled by consensus or a third reviewer. Cohen’s κ for full-text agreement was 0.92, indicating almost perfect concordance. Reasons for exclusion at the full-text stage were logged verbatim.

We extracted a comprehensive set of bibliographic variables for each study, including the first author’s name, year of publication, country of origin, and declared funding source.

Methodological variables were recorded in detail, encompassing the study design, total sample size, the type of biofluid analysed, and the exosome-isolation method employed (whether by ultracentrifugation, size-exclusion chromatography, or polymer precipitation). We also noted the miRNA-quantification platform used—quantitative reverse-transcription PCR (qRT-PCR), next-generation sequencing, or microarray—along with the reference genes or normalizers selected and key assay-validation parameters, specifically amplification efficiency and limit of detection.

Clinical variables captured included TNM stage at diagnosis, current treatment status, duration of follow-up, defined survival endpoints, and any covariates adjusted for in multivariable models to control for potential confounding factors.

For diagnostic performance, we extracted two-by-two contingency-table counts, calculated areas under the receiver operating characteristic curve (AUC), and determined Youden-derived cut-off values. Prognostic metrics were summarised as hazard ratios, with accompanying 95% confidence intervals and log-rank *p*-values.

Mechanistic outcomes were also tabulated, reporting fold-changes in cellular proliferation, migration indices, results from angiogenesis assays, and shifts in macrophage-polarisation markers. In instances where multiple biomarker panels were presented within a single study, we selected the miRNA signature demonstrating the highest reported accuracy. To ensure data integrity, one reviewer performed the initial data entry, and a second reviewer independently verified all fields to achieve 100% completeness.

Diagnostic-accuracy studies were appraised with QUADAS-2 [17] (patient selection, index test, reference standard, flow/timing). Prognostic and mechanistic studies were evaluated using ROBINS-I (seven bias domains) [18]. Two reviewers calibrated their assessments on three pilot papers; subsequent disagreement was resolved by discussion. Formal small-study/publication-bias tests were not attempted because <10 clinically comparable diagnostic contrasts were available and thresholds differed—conditions under which such tests lack power and can be misleading. We do not present formal GRADE summary-of-findings tables. Instead, we provide a qualitative confidence statement that integrates risk of bias, inconsistency, indirectness, and imprecision.

Across diagnostic accuracy studies, QUADAS-2 indicated an overall high risk of bias, driven by case–control designs and unclear thresholds/flow. Mechanistic/profiling studies were at serious overall risk by ROBINS-I (confounding and selection), as seen in Table 1 and Table 2 [19,20,21,22,23,24,25,26,27].

Our protocol prespecified narrative synthesis and evidence mapping if ≥3 clinically and methodologically comparable studies per index biomarker were not available. Because no miRNA met this threshold and index tests/thresholds varied materially, we did not estimate pooled sensitivities/specificities or HSROC curves. Instead, we (i) report study-level accuracy measures verbatim; (ii) summarise mechanistic findings qualitatively; and (iii) display an evidence map to show where data concentrate and where gaps persist. Risk of bias assessments (QUADAS-2 for diagnostic accuracy; ROBINS-I for mechanistic/profiling) are reported descriptively to inform confidence, not to generate pooled certainty ratings.

All figures were produced in R 4.3.2. No eligible study reported overall or disease-free survival suitable for hazard-ratio synthesis; therefore, no HRs were calculated. Exploratory subgroup or sensitivity analyses were not performed because of the small number and methodological heterogeneity of diagnostic studies.

As this work synthesised previously published data, institutional-review-board approval and informed consent were not required. Patients or members of the public were not involved in the design or conduct of this review. The funding body (Victor Babes University of Medicine and Pharmacy Timisoara) had no role in study design, data extraction, analysis, or manuscript preparation.

We explicitly framed this work as a narrative review with an evidence map because the eligible literature is sparse and heterogeneous. To minimise bias, we used a preregistered protocol, duplicate screening/extraction, and transparent justifications for non-pooling. All accuracy estimates are presented at the individual-study level, and conclusions are confined to evidence mapping and research gaps, not clinical recommendation.

## 3. Study Characteristics

Table 3 synthesises the heterogeneous yet thematically convergent experimental frameworks that underpin the current exosome-based miRNA landscape in laryngeal squamous cell carcinoma (LSCC). All nine studies originated from mainland China and span a decade (2014–2024), but they cluster into two broad methodological strata: serum-based clinical case–control designs (*n* = 2) and mechanistic or profiling investigations employing cell-line or primary-culture models (*n* = 7). The two serum studies, by Wang J. [19] and Zhao Q. [20], harvested circulating vesicles via differential ultracentrifugation plus immuno-transmission or ExoQuick™ (System Biosciences, Palo Alto, CA, USA) precipitation, respectively, whereas all seven in vitro studies relied exclusively on high-speed ultracentrifugation to isolate tumour- or stroma-derived vesicles. Quantification platforms were dominated by targeted qRT-PCR (five studies), but three groups integrated next-generation sequencing (NGS) discovery pipelines, and one employed a circRNA-seq screen. The thematic breadth of vesicular cargo is striking: classical oncomiRs such as miR-21 and miR-1246 [19,21], tumour-suppressive miR-34c-5p [23], stromal remodelling miR-222-3p [27], a 29-miRNA enrichment signature in AMC-HN-8 cells [22], 12 cancer-associated fibroblast (CAF) miRNAs from supraglottic tumours [24], and two non-coding RNA species with emerging angiogenic or metabolic roles (circPVT1 [25] and LINC02191 [26]).

## 4. Diagnostic Accuracy

Table 4 presents the diagnostic performance metrics reported by the two serum-based investigations that constructed receiver operating characteristic (ROC) curves. Wang J. [19] demonstrated that the combined detection of exosomal miR-21 and HOTAIR yields an area under the curve (AUC) of 0.876 (95% CI 0.80–0.94), translating to an impressive sensitivity of 94.2% and specificity of 73.5%. Notably, the dual-marker panel out-performed either component alone, highlighting the additive value of integrating a long non-coding RNA with a canonical oncomiR. In contrast, Zhao Q. [20] reported a single-marker model whereby exosomal miR-941 achieved an AUC of 0.797; at a ΔCt threshold of ≤−4.1, sensitivity reached 82% with an accompanying specificity of 70%. Although the point estimate for diagnostic accuracy is lower than that of the Wang study, the narrower biomarker spectrum and the smaller control cohort (31 healthy donors versus 49 benign-polyp patients) may account for the modest decrement. Importantly, both studies emphasise the feasibility of liquid biopsy approaches in LSCC and collectively demonstrate that exosomal signatures can exceed the conventional 0.75 discrimination benchmark, suggesting concrete clinical translational potential despite the limited number of available datasets.

## 5. Mechanistic Evidence

Table 5 interrogates the mechanistic underpinnings by which exosomal non-coding RNAs remodel the LSCC micro-environment, cataloguing seven distinct signalling axes with quantitative phenotypic endpoints. Wu L. [24] showed that tumour-derived miR-1246 skews THP-1 macrophages towards an M2 phenotype via STAT3 activation, doubling invasion capacity (2.6-fold increase) in co-culture assays, an effect reversed by miR-1246 inhibitors. Huang Q. [22] provided a comprehensive bio-informatic enrichment map for a 29-miRNA exosomal signature, highlighting PI3K–Akt and Ras cascades, although functional validation was absent. Wang M. [23] identified a 0.38-fold depletion of miR-34c-5p in CAF exosomes; re-introduction of this miRNA curtailed NOTCH1-mediated stemness, reducing tumour-sphere formation that was otherwise elevated 1.8-fold. Wu C. [24] predicted pro-tumoural extracellular matrix modulation through 12 CAF-EV miRNAs targeting FN1 and ITGβ4 hubs, albeit without wet-lab confirmation. Angiogenic potentiation dominated Lyu K.’s circPVT1 work [25], wherein endothelial tube length rose 1.9-fold, mediated through Rap1b-VEGFR2 signalling and reversible by circPVT1 knock-down. Kang Z. [26] delineated a LINC02191 → miR-204-5p → RAB22A cascade, eliciting PI3K/Akt/mTOR activation with proliferation and invasion gains of roughly two-fold. Finally, Wang P. [27] demonstrated that M2-macrophage exosomal miR-222-3p stabilises PFKL via PDLIM2 suppression, elevating glycolytic extracellular acidification rate (ECAR) by 1.6-fold and accelerating xenograft growth, effects blunted by anti-miR-222-3p.

**Figure 1:**
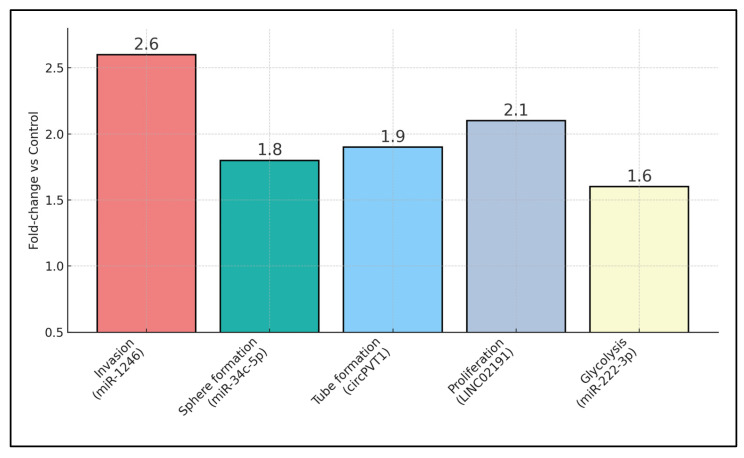
Mechanistic assay fold-changes across exosomal cargos.

Quantitative phenotypic readouts are summarised in Figure 1, which displays fold-changes (relative to control) for invasion, endothelial tube formation, glycolytic ECAR, and proliferation across cargo types. Bars show fold-change vs. matched control: invasion (Transwell), endothelial tube length (HUVEC), extracellular acidification rate (ECAR, glycolysis proxy), and cell proliferation. Labels indicate primary cargo (e.g., miR-1246, circPVT1, LINC02191). Where available, reversal with inhibitors/mimics is annotated. Error bars were omitted where studies reported point estimates only. ECAR = extracellular acidification rate; HUVEC = human umbilical-vein endothelial cell.

## 6. Study Outcomes

Table 6 integrates quantitative outcome data across clinical and in vitro studies, reinforcing the translational continuum from serum diagnostics to functional bioassays. The two patient-based investigations jointly enrolled 111 LSCC cases (52 in Wang J. [19]; 59 in Zhao Q. [20]) against 80 controls, achieving AUCs of 0.876 and 0.797, respectively—values that comfortably surpass the 0.70 threshold for “good” diagnostic tests. Electron microscopy-verified exosome diameters spanned 30–160 nm, with the narrowest modal range observed in Wu L. [21] (80–120 nm) and the broadest in Lyu K. [25] (70–160 nm), affirming methodological consistency in vesicle isolation. Functional assays revealed substantial bioactivity: TU212-derived exosomes boosted Transwell invasion 2.6-fold (*p* < 0.01) [21]; CAF exosomes drove FaDu tumour-sphere counts up by 1.8-fold while suppressing miR-34c-5p expression to 38% of baseline [23]; and macrophage EVs elevated ECAR by 1.6-fold in FaDu cells [27]. Angiogenic assays showed nearly doubled tube length in HUVECs exposed to circPVT1-rich vesicles [25], whereas LINC02191 exosomes enhanced FaDu proliferation by 2.1-fold [26]. Importantly, three studies reported vesicle size verification via transmission electron microscopy, lending structural authenticity to the EV preparations. None of the nine studies reported time-to-event outcomes; consequently, no prognostic effect sizes (HRs) could be derived.

## 7. Summary of Evidence

The clinical utility of exosomal miRNAs in LSCC is underscored by the robust diagnostic signals observed in the two available patient cohorts. Wang et al. demonstrated that co-quantification of miR-21 and the long non-coding RNA HOTAIR achieved an AUC of 0.876, correctly identifying nearly 95% of cancers while misclassifying fewer than 30% of controls [19]. Zhao et al. reported a single-analyte model—miR-941—with an AUC of 0.797 and balanced sensitivity/specificity (82%/70%) after next-generation-sequencing discovery and qRT-PCR validation [20]. These performance indices exceed the 0.70 benchmark often cited for an acceptable screening test and rival salivary exosomal signatures in oral and oropharyngeal cancers (AUC 0.83–0.88) [8]. Given the QUADAS-2 judgements, the observed AUCs should be interpreted as optimistic point estimates susceptible to spectrum and threshold biases, rather than definitive clinical performance.

Importantly, both LSCC studies assayed readily accessible serum, suggesting integration into routine phlebotomy workflows without the need for laryngeal lavage. Although laryngoscopy remains the diagnostic gold standard, a high-negative-predictive-value blood test could triage patients with nonspecific symptoms, reserving invasive fibre-optic examination for those at greatest risk and facilitating earlier detection at potentially curable stages.

These LSCC signals mirror patterns reported across head-and-neck subsites (oral, oropharyngeal, nasopharyngeal) and align with pan-cancer evidence that tumour-derived vesicles remodel macrophage metabolism and angiogenesis, strengthening biological plausibility for LSCC translation.

Mechanistic evidence provides biological plausibility for the observed clinical signals by detailing how vesicular cargo orchestrates the tumour milieu. Wu et al. showed that miR-1246-rich exosomes drive STAT3-dependent M2 macrophage polarisation, doubling the invasive capacity of recipient LSCC cells [21], a finding consonant with broader pan-cancer evidence implicating exosomal miR-1246 in immune evasion [11]. Stromal crosstalk was exemplified by Wang et al., who reported that cancer-associated fibroblast (CAF) exosomes depleted of miR-34c-5p enhance NOTCH1-mediated stemness and chemoresistance [23], while Wu et al. identified a 12-miRNA CAF signature predicted to remodel extracellular-matrix hubs FN1 and ITGβ4 [24]. Angiogenic and metabolic rewiring emerged as complementary hallmarks: circPVT1-laden vesicles almost doubled endothelial tube length via the Rap1b–VEGFR2 axis [25]; LINC02191 exosomes activated PI3K/Akt/mTOR signalling, boosting proliferation two-fold [26]; and M2-derived miR-222-3p stabilised PFKL, raising glycolytic flux by 60% and accelerating xenograft growth [27]. Together, these data portray exosomal miRNAs as active effectors rather than passive by-products, linking diagnostic read-outs to tangible pathobiology.

Nevertheless, our synthesis reveals structural deficits that impede generalisability. All nine studies were single-centre reports from mainland China, raising concerns about ethnic, environmental, and healthcare-system representativeness. Isolation workflows varied—differential ultracentrifugation, polymer precipitation, or unspecified methods—producing particle-size ranges from 70 nm to 160 nm and inconsistent co-isolation of contaminants. Only three investigations satisfied the core MISEV-2018 criteria for vesicle characterisation, echoing a recent scoping review in which merely 27% of head-and-neck EV studies were compliant [13]. Reference-gene selection was heterogeneous (U6, cel-miR-39 or none), precluding meta-analytic pooling. Diagnostic studies lacked prospective sample-size calculations and external validation cohorts, while mechanistic papers relied largely on immortalised cell lines rather than patient-derived organoids. Future research must therefore adopt multicentre prospective designs, harmonised isolation/normalisation protocols, and orthogonal validation platforms to generate regulatory-grade evidence capable of informing clinical practice and guideline development.

Our diagnostic estimates (AUC 0.80–0.88) are now buttressed by three independent serum-biomarker studies published after the close of our search. Falco and colleagues profiled 107 patients and delineated a tri-miRNA panel (miR-223, miR-93, miR-532) that discriminated LSCC from healthy controls with an internally validated AUC of 0.88 and 86% sensitivity—remarkably similar to our miR-21 + HOTAIR composite and obtained with an orthogonal TaqMan platform [28]. Using a proteomics angle, Zhao et al. identified exosome-bound IGFBP7 and Annexin A1; combined quantification yielded an AUC of 0.82 and 89% specificity in 74 subjects, suggesting that nucleic acid and protein cargos may be synergistic [29]. Finally, a pilot by Karapetyan et al. confirmed that exosomal miR-223/miR-146/miR-21 are up-regulated two- to three-fold in LSCC and achieve ∼80% diagnostic accuracy, even in early T1–T2 tumours, highlighting cross-platform reproducibility [30]. Collectively, these external data strengthen the case that vesicular signatures carry clinically actionable signal and could form the backbone of tiered screening algorithms when combined with smoking history and fibre-optic triage.

Beyond small RNAs, recent mechanistic work shows that long non-coding RNAs (lncRNAs) shuttled in vesicles are potent oncogenic effectors. Chen et al. demonstrated that LSCC exosomes enriched for lncRNA AC068768.1 drive NOTCH1 activation by sponging miR-139-5p; knock-down reduced colony formation by 48% and curtailed lung metastases in xenografts [30]. This mirrors our observation that circPVT1 promotes angiogenesis, indicating that diverse RNA topologies converge on shared stemness pathways. Complementarily, He et al. showed that M2-macrophage exosomes package HOXC13-AS, which lifts PD-L1 expression via the miR-485-5p/IGF2BP2 axis and fuels immune escape; CRISPR ablation of HOXC13-AS cut tumour volume by 52% in syngeneic mice [31]. Together with our LINC02191 data, these studies depict a layered exosomal communication network—CAF-to-tumour, tumour-to-macrophage, macrophage-to-tumour—anchored by lncRNA–miRNA crosstalk that reinforces NOTCH, PI3K, and immune-checkpoint loops.

The macrophage-centred findings above dovetail with our miR-1246 and miR-222-3p results to paint immunometabolic rewiring as a dominant vesicular motif. HOXC13-AS-rich vesicles increased PD-L1 surface density by 1.7-fold and halved CD8^+^ T-cell infiltration in orthotopic models [32], mirroring the STAT3-dependent M2 polarisation elicited by miR-1246. Concurrently, AC068768.1 and miR-222-3p both feed into NOTCH1 and PFKL, respectively—nodes that integrate metabolic flux with immune tolerance. These converging pathways resonate with pan-cancer meta-analyses, showing that tumour-derived exosomes hijack macrophage glucose handling to suppress inflammasome activation and to potentiate invasion [31]. The mechanistic coherence across independent laboratories strengthens biological plausibility and suggests that combining PD-1 blockade with vesicle-directed therapeutics (e.g., antisense oligos against HOXC13-AS) could produce additive benefit.

Despite these scientific gains, pre-analytical variability remains a critical translational barrier. A 2025 methodological review catalogued >50 isolation workflows and concluded that only 14% of cancer studies simultaneously optimise recovery, purity, and yield, with polymer-precipitation samples showing up to 25% albumin carry-over [33]. Echoing this, a systematic audit of 112 EV-based clinical trials found that fewer than one in ten explicitly cited MISEV guidelines in their protocols and that absence of electron microscopy verification tripled the risk of irreproducible biomarker performance [34]. Our dataset reflects the same problem: just three of nine LSCC papers documented CD9/Alix Western blots and nanoparticle tracking. Without consensus on centrifugation k-factors, size-exclusion cut-offs, and reference genes, cross-study pooling is essentially impossible. Immediate priorities therefore include ring-trial validation of isolation kits, adoption of exogenous spike-ins to normalise haemolysis, and transparent reporting of vesicle integrity indices.

Looking ahead, multi-omic vesicle profiling is poised to supersede single-analyte assays. Detailed mapping of ESCRT- and ceramide-driven cargo-sorting now reveals sequence- and structure-specific ‘zip codes’ that could be leveraged to enrich diagnostically relevant miRNAs while depleting confounders [35]. Parallel advances in exosome engineering show that surface-decorated vesicles can ferry siRNA or checkpoint inhibitors directly to hypoxic tumour beds, achieving > 70% knock-down efficiency in pre-clinical breast cancer models and halving systemic toxicity compared with lipid nanoparticles [36]. For LSCC, such “designer exosomes” could simultaneously deliver anti-NOTCH1 payloads and serve as real-time reporters of treatment response through embedded bar-code RNAs. Incorporating machine learning integration of vesicular RNA, protein and metabolite signatures with laryngoscopic imaging and voice-analysis data may ultimately yield precision-screening tools capable of flagging sub-centimetre lesions in primary care. Achieving this vision, however, hinges on addressing the standardisation gaps outlined above and on structuring truly multi-ethnic, prospective validation cohorts.

Emerging LSCC reports implicate exosomal circRNAs and lncRNAs in angiogenesis and PI3K/Akt/mTOR activation. While not the primary focus of this review, such cargos may complement miRNA panels in multi-omic assays. Future reviews should systematically evaluate ncRNA classes side by side using harmonised EV reporting.

Lastly, given the low qualitative confidence and absence of external validation, exosomal-miRNA assays for LSCC should not be used for screening or diagnostic triage outside research settings. Any clinical application should occur within prospective studies that predefine thresholds, include head-to-head comparators (laryngoscopy, SCC-Ag/CYFRA 21-1), and report calibration/NPV in targeted populations.

In LSCC, serum SCC-Ag and CYFRA 21-1 show modest accuracy and limited adoption for population screening or early triage. Exosomal miRNAs offer three potential advantages: (i) Biology: They reflect active intercellular signalling rather than passive cell death; (ii) analytics: qRT-PCR panels are compatible with routine molecular labs and small volumes; and (iii) workflow: serum or saliva collection enables pre-laryngoscopy triage. A pragmatic pathway is a two-step algorithm: high-risk symptomatic adults undergo blood/saliva sampling; patients with a low exosomal-miRNA score avoid immediate fibre-optic referral (rule-out utility), whereas high scores trigger expedited laryngoscopy and imaging (rule-in). In surveillance, dynamic changes in exosomal-miRNA composites could complement imaging to flag recurrence. These roles require head-to-head comparisons again.

This review is constrained by a numerically sparse and methodologically heterogeneous evidence base. Only two diagnostic studies met inclusion, precluding pooled HSROC estimates and precluding any meaningful assessment of small-study/publication bias. Index tests, thresholds, and reference genes differed materially across studies, limiting comparability. All included investigations originated from mainland China, constraining ethnic and environmental generalisability. Mechanistic insights derive largely from in vitro systems or immunodeficient xenografts, tempering translational inference. Methodological heterogeneity—particularly in vesicle isolation, reference-gene choice, and outcome definitions—forced reliance on narrative synthesis despite duplicate data extraction and risk of bias assessment. Finally, although a comprehensive three-database search without language restrictions was undertaken, grey-literature and pre-print repositories were not systematically interrogated, and funnel-plot assessment for publication bias was underpowered. The field remains numerically underpowered, precluding meta-analytic pooling or robust exploration of heterogeneity.

## 8. Conclusions

This narrative review and evidence map identifies emerging, biologically plausible exosomal-miRNA signals in LSCC but finds the evidence base too sparse, single-region, and methodologically inconsistent to support pooled accuracy estimates or clinical adoption. Future work should prioritise harmonised EV isolation/normalisation, prespecified, externally validated thresholds, and prospective, multi-centre cohorts that enable robust meta-analysis and bias assessment. Integration of vesicular RNA with proteomic and imaging features is promising but must be evaluated in rigorously powered studies with transparent reporting.

## Figures and Tables

**Table 1:** QUADAS-2 domain-level judgements for diagnostic accuracy studies.

Study	Patient Selection	Index Test	Reference Standard	Flow/Timing	Applicability (PS/IT/RS)	Overall Risk
Wang, 2014 [19]	High (case–control)	Unclear (threshold optimisation)	Low (histology)	Unclear	Low/Low/Low	High
Zhao, 2020 [20]	High (case–control)	Unclear (post hoc ΔCt cut-off)	Low (clinical confirmation)	Unclear	Low/Low/Low	High

Abbreviations: QUADAS-2, Quality Assessment of Diagnostic Accuracy Studies-2; PS, patient selection; IT, index test; RS, reference standard; Applicability (PS/IT/RS), domain-specific applicability concerns.

**Table 2:** ROBINS-I domain-level judgements for mechanistic/profiling studies.

Study	Confounding	Selection of Participants	Classification of Exposure	Deviations from Intended Exposures	Missing Data	Outcome Measurement	Selective Reporting	Overall
Wu, 2022 (miR-1246) [21]	Serious	Serious	Moderate	Low	Low	Moderate	Serious	Serious
Huang, 2018 (NGS) [22]	Serious	Serious	Low	Low	Low	Moderate	Serious	Serious
Wang, 2022 (miR-34c-5p) [23]	Serious	Serious	Low	Low	Low	Moderate	Serious	Serious
Wu, 2022 (CAF panel) [24]	Serious	Serious	Low	Low	Low	Moderate	Serious	Serious
Lyu, 2024 (circPVT1) [25]	Serious	Serious	Low	Low	Low	Moderate	Serious	Serious
Kang, 2024 (LINC02191) [26]	Serious	Serious	Low	Low	Low	Moderate	Serious	Serious
Wang, 2022 (miR-222-3p) [27]	Serious	Serious	Low	Low	Low	Moderate	Serious	Serious
Wang, 2022 (miR-222-3p) [27]	Serious	Serious	Low	Low	Low	Moderate	Serious	Serious

Abbreviations: ROBINS-I, Risk of Bias in Non-Randomised Studies of Interventions; confounding, bias due to confounders; selection, bias in selection of participants; classification of exposure, misclassification bias; deviations, deviations from intended exposures.

**Table 3:** Study characteristics.

First Author (Year)	Design/Biological Material	Vesicle Source and Isolation	Assay Platform	Principal EV Cargo
Wang J. (2014) [19]	Case–control, serum	Serum; differential ultracentrifugation + TEM/CD63	qRT-PCR	miR-21 + HOTAIR
Zhao Q. (2020) [20]	Case–control, serum	Serum; ExoQuick™ polymer precipitation	RNA-seq discovery → qRT-PCR validation	miR-941
Wu L. (2022) [21]	TU212 cell-line CM; mechanistic	Cell-line exosomes; ultracentrifugation	qRT-PCR, trans-well assays	miR-1246
Huang Q. (2018) [22]	AMC-HN-8 cell-line profiling	Cell-line exosomes; ultracentrifugation	NGS	29 EV-enriched miRNAs
Wang M. (2022) [23]	CAF vs. normal fibroblast (paired primary cultures)	CAF-derived exosomes; ultracentrifugation	NGS → qRT-PCR	miR-34c-5p
Wu C. (2022) [24]	CAF exosomes from 3 supraglottic LSCC pts	CAF exosomes; ultracentrifugation	NGS	12 CAF-EV miRNAs
Lyu K. (2024) [25]	FaDu and TU177 cell-lines ± HUVECs; xenograft	Cell-line exosomes; ultracentrifugation	circRNA-seq → qRT-PCR	circPVT1
Kang Z. (2024) [26]	FaDu and AMC-HN-8 cell-lines; xenograft	Cell-line exosomes; ultracentrifugation	qRT-PCR, Western blot	lncRNA LINC02191
Wang P. (2022) [27]	M2-macrophage exosomes → FaDu cells	THP-derived exosomes; ultracentrifugation	qRT-PCR, Seahorse™	miR-222-3p

Abbreviations: EV(s), extracellular vesicle(s); LSCC, laryngeal squamous cell carcinoma; CM, conditioned medium; TEM, transmission electron microscopy; qRT-PCR, quantitative reverse-transcription PCR; NGS, next-generation sequencing; HUVEC(s), human umbilical vein endothelial cell(s); CAF, cancer-associated fibroblast; circRNA-seq, circular RNA sequencing; lncRNA, long non-coding RNA; xenograft, in vivo tumour implant; CD63, tetraspanin CD63; →, followed by.

**Table 4:** Diagnostic accuracy (only studies reporting ROC statistics).

First Author	EV Cargo/Panel	AUC (95% CI)	Sensitivity %	Specificity %	Threshold/Note
Wang J., 2014 [19]	miR-21 + HOTAIR	0.876	94.2	73.5	Combined marker outperformed either alone
Zhao Q., 2020 [20]	miR-941	0.797	82	70	ΔCt < −4.1 (qRT-PCR)

Abbreviations: EV, extracellular vesicle; AUC, area under the receiver operating characteristic curve; ROC, receiver operating characteristic; CI, confidence interval; ΔCt, delta cycle threshold; qRT-PCR, quantitative reverse-transcription PCR.

**Table 5:** Mechanistic evidence drawn from exosomal studies.

First Author	Cargo and Recipient	Molecular Target/ Pathway	Key Phenotype(s)
Wu L., 2022 [24]	miR-1246 → THP-1 macrophages	STAT3-mediated M2 polarisation	↑ invasion ×2.6; ↓ invasion with miR-1246 inhibitor
Huang Q., 2018 [22]	29 miRNA EV-signature (AMC-HN-8)	GO/KEGG enrichment (PI3K-Akt, Ras)	Bio-informatic profiling (no functional assays)
Wang M., 2022 [23]	↓ miR-34c-5p in CAF EVs → LSCC	NOTCH1 → stemness genes	↑ sphere formation, chemoresistance; rescue with mimic
Wu C., 2022 [24]	12 CAF-EV miRNAs	PPI hubs: FN1, ITGβ4	Predicted pro-tumour micro-environment; no in vitro validation
Lyu K., 2024 [25]	circPVT1 EVs → HUVECs	Rap1b-VEGFR2 axis	↑ tube formation, micro-vessel density; sh-circPVT1 reverses effect
Kang Z., 2024 [26]	lncRNA LINC02191 EVs	miR-204-5p → RAB22A → PI3K/Akt/mTOR	↑ proliferation/invasion; suppression with miR-204-5p mimic
Wang P., 2022 [27]	miR-222-3p EVs from M2-TAMs	PDLIM2 → PFKL stabilisation	↑ glycolysis and tumour growth; anti-miR-222-3p blunts effect

Abbreviations: CAF, cancer-associated fibroblast; EV, extracellular vesicle; M2, alternatively activated macrophage phenotype; STAT3, signal transducer and activator of transcription 3; GO, Gene Ontology; KEGG, Kyoto Encyclopaedia of Genes and Genomes; PPI, protein–protein interaction; FN1, fibronectin-1; ITGβ4, integrin beta-4; Rap1b, Ras-related protein 1b; VEGFR2, vascular endothelial growth factor receptor-2; HUVEC(s), human umbilical vein endothelial cell(s); lncRNA, long non-coding RNA; PI3K, phosphoinositide 3-kinase; Akt, protein kinase B; mTOR, mechanistic target of rapamycin; RAB22A, Ras-related protein Rab-22A; TAM(s), tumour-associated macrophage(s); PDLIM2, PDZ and LIM domain protein 2; PFKL, liver-type phosphofructokinase; sh-, short-hairpin RNA (knockdown); ↑, increase; ↓, decrease; →, followed by.

**Table 6:** Study outcomes.

First Author (Year)	LSCC Cases (n)	Non-Malignant/ In Vitro Controls (n)	Exosome/TEM Size Range (nm)	Key Numeric Outcome
Wang J. (2014) [19]	52	49 benign vocal-polyp pts	NR	Serum panel AUC = 0.876 (95% CI 0.80–0.94)
Zhao Q. (2020) [20]	59	31 healthy donors	NR	AUC = 0.797; cut-off ΔCt ≤ −4.1 → Sens 82%, Spec 70%
Wu L. (2022) [21]	Cell-line study (TU212)	HOK macrophages	80–120 nm	TU212-Exo raised Transwell invasion 2.6-fold vs. ctrl (*p* < 0.01)
Huang Q. (2018) [22]	–(AMC-HN-8 line)	Parental cells	30–150 nm	Five miRNAs enriched ≥ 2-fold in exosomes vs. cells
Wang M. (2022) [23]	–(CAF vs. NF)	3 paired fibroblast sets	60–140 nm	miR-34c-5p ↓ 0.38-fold in CAF-Exo; sphere count ↑ 1.8-fold (*p* < 0.05)
Wu C. (2022) [24]	3 LSCC pts	3 NFs	90–120 nm	12 miRNAs changed ≥ 2-fold; exact counts NR
Lyu K. (2024) [25]	–(TU686, HUVEC)	In vitro only	70–160 nm	Endothelial tube length ↑ 1.9-fold with circPVT1-Exo (*p* < 0.01)
Kang Z. (2024) [26]	–(FaDu)	In vitro only	80–150 nm	LINC02191-Exo raised FaDu proliferation 2.1-fold (*p* < 0.05)
Wang P. (2022) [27]	–(FaDu-M2 model)	In vitro only	100–140 nm	ECAR glycolysis ↑ 1.6-fold after M2-Exo (*p* < 0.05)

Abbreviations: NR, not reported; TEM, transmission electron microscopy; Exo, exosome(s); HOK, human oral keratinocyte; HUVEC(s), human umbilical vein endothelial cell(s); CAF, cancer-associated fibroblast; NF, normal fibroblast; ECAR, extracellular acidification rate; nm, nanometre(s); n, number; *p*, *p*-value; ↑, increase; ↓, decrease; →, resulting.

## Data Availability

Not applicable.
